# Developing HIV risk prediction tools in four African settings

**DOI:** 10.1111/tmi.13916

**Published:** 2023-07-27

**Authors:** Sheila Kansiime, Christian Holm Hansen, Richard Hayes, Eugene Ruzagira, the PrEPVacc Study Team

**Affiliations:** ^1^ Medical Research Council/Uganda Virus Research Council and London School of Hygiene and Tropical Medicine, Uganda Research Unit Entebbe Uganda; ^2^ Medical Research Council International Statistics and Epidemiology Group, London School of Hygiene & Tropical Medicine London UK

**Keywords:** HIV incidence, HIV prevalence, HIV risk score, HIV/AIDS

## Abstract

**Objective:**

HIV risk prediction tools are a critical component of efforts to end the HIV pandemic. We aimed to create and validate tools for identifying individuals at highest risk of prevalent and incident HIV in an African setting.

**Methods:**

We used Logistic regression and Poisson regression to determine risk factors for HIV prevalence and incidence in a multi‐country HIV vaccine trial preparedness cohort study among individuals at high risk of HIV, and used the identified factors to create and validate tools that predict HIV risk. We also assessed the performance of the VOICE risk score in predicting HIV incidence among women in the cohort.

**Results:**

The prevalent HIV prediction tool created had good predictive ability [area under the curve (AUC) = 0.70, 95% CI 0.66–0.74]. It included the following participant variables: age, sex, recreational drug use, unprotected male‐to‐male anal sex, a sexual partner who had other partners, transactional sex and having a partner who was a long‐distance truck driver/miner. It was not possible to create a valid HIV incidence prediction tool. Participants with high VOICE risk scores (≥7) had slightly higher HIV incidence but this tool performed poorly within our study (AUC = 0.58, 95% CI 0.51–0.64: Harrell's concordance index = 0.59).

**Conclusion:**

We created a prevalent HIV prediction tool that could be used to increase efficiency in diagnosis of HIV and linkage to care in sub‐Saharan Africa. Existing incident HIV prediction tools may need modification to include context‐specific predictors such as calendar period, participant occupation, study site, before adoption in settings different from those in which they were developed.

## INTRODUCTION

HIV incidence has reduced world‐wide but remains high in particular settings [1]. For sub‐Saharan Africa (SSA) which accounts for almost 60% of the global burden of HIV infections [2, 3], the UNAIDS goal of “ending AIDS” by 2030 will be difficult to attain despite progress being made elsewhere [4].

Advances have been made in developing effective HIV prevention interventions over the last few years [5]. However, limited progress has been made in ensuring that these interventions are accessible and affordable. Uptake of HIV pre‐exposure prophylaxis (PrEP) in SSA has also been challenged by social barriers and misconceptions [6].

Low HIV risk perception, even among persons self‐reporting high risk behaviours, is one of the hindrances to the uptake of interventions such as PrEP and condoms [6]. HIV risk prediction tools that identify individuals at highest risk of HIV infection could be useful in this regard.

Risk prediction tools that identify individuals likely to be HIV infected could be used to target testing and linkage to care more efficiently. This would increase awareness of HIV positive status and antiretroviral therapy (ART) coverage, moving the world closer to achieving the 95‐95‐95 UNAIDS targets. In SSA, HIV prevalence prediction tools are scarce [7] despite the potential benefit they could have for HIV Treatment as Prevention efforts as seen with the Denver prevalence risk score in the United States of America [8].

Furthermore, an HIV incidence risk score that identifies individuals at high risk and predicts short term HIV incidence would be useful in identifying study populations for HIV prevention trials and for targeting HIV prevention interventions [9, 10].

Several HIV incidence risk scores have been created in SSA [7, 11, 12]. The VOICE risk score is a 1‐year HIV risk prediction tool which was shown to have reasonable predictive ability [AUC estimated at 0.71 (95% CI: 0.68–0.74)] among women at high risk in eastern and southern Africa. It was developed using data from the VOICE HIV prevention clinical trial (NCT00705679) conducted 2009–2012 [13]. It continues to be used for risk assessments and in recruitment for HIV prevention trials several years later [14, 15]. Assessments of its performance and generalisability across various settings in Africa over time are limited.

Using data from a multi‐country HIV vaccine trial preparedness study, the PrEPVacc registration cohort [16], we sought to identify indicators most predictive of prevalent and incident HIV infection at screening and during follow‐up respectively; and thereafter create and validate risk prediction tools that could identify individuals at high risk of HIV. We also assessed the performance of the VOICE HIV risk score tool in predicting HIV incidence among women in the cohort within their first year of follow‐up.

## MATERIALS AND METHODS

### Study design and population

This study is based on screening and follow‐up data from the PrEPVacc registration cohort that is being undertaken in four African countries in preparation for the PrEPVacc trial, a Phase IIb three‐arm, two‐stage HIV prophylactic vaccine trial with a second randomisation to compare TAF/FTC to TDF/FTC as pre‐exposure prophylaxis (NCT04066881) [16].

The target population for the PrEPVacc registration cohort study comprised adults (18–45 years) at high‐risk of HIV infection, that is, female sex workers (FSW), and female and male fisher folk in Masaka, Uganda; female bar workers and FSW in Dar es Salaam and Mbeya, Tanzania; men who have sex with men (MSM), FSW and other at risk individuals from the general population in Maputo, Mozambique; and the general population in areas of known high HIV incidence in Durban, South Africa [17].

Potential participants were invited and provided with detailed information on study inclusion/exclusion criteria (including being HIV negative) and other study procedures. At screening, individuals were determined to be eligible if they were confirmed to be HIV‐uninfected, considered to be at risk of HIV infection through initial pre‐screening conversations, willing to provide informed consent, undergo regular HIV testing and counselling (HCT), undergo regular pregnancy testing if female, provide adequate locator information, and complete interviewer‐administered questionnaires on behavioural and other HIV risk factors.

At enrolment and quarterly visits, individuals received HCT and for those that tested HIV‐negative, counselling on and referrals for PrEP where it was available [16, 18]. Oral Truvada (TDF/FTC) was available on site in Durban and through referral to a local provider in Masaka from 2018 when the PrEPVacc registration cohort was initiated. PrEP became available through referral to local providers in Mbeya and Dar es Salaam early—mid 2021. PrEP was not available in Maputo. PrEP uptake during follow up was monitored through self‐report every 6 months.

### HIV diagnosis

HIV testing was conducted in accordance with site‐specific algorithms and national HIV testing guidelines. HIV infection was defined as the detection of HIV specific antibodies by at least two different HIV antibody tests. Individuals who tested HIV‐positive were referred to their preferred health care facility and followed up to ensure linkage to care. Free HIV care services are available across all our study settings.

### Statistical analysis

Data analysis was conducted in Stata version 16.0 (College Station, TX, US). Key predictors of HIV risk were identified from literature and investigated within the registration cohort data (data as of 16/11/2021) (key variables are provided in Table S1).

### Analysis of HIV prevalence, incidence, and associated risk factors

We assessed associations between baseline socio‐demographic and behavioural risk characteristics, and HIV prevalence at screening using univariable and multivariable Logistic regression. Associations with HIV incidence during follow‐up were assessed using univariable and multivariable Poisson regression models. Forward stepwise model building was used to derive the final models with predictors added to the model if they had a *p* value < 0.2. Study site was included in the models a priori. Goodness of fit of the final logistic regression model was assessed using the Hosmer‐Lemeshow goodness of fit test.

HIV incidence was assumed to occur at the midpoint between the dates of the last negative and first positive HIV test results. Associations between follow‐up time and HIV incidence were also assessed using Lexis expansions to create categories for time in follow‐up (first year, second year, third year or higher) and calendar period (2018, 2019, 2020/2021). The 2020 and 2021 calendar periods were combined due to the limited number of person years in 2021.

### HIV prevalence and incidence prediction models

A similar approach to that used for the development and validation of HIV incidence risk scores among MSM in the US and HIV sero‐discordant couples and women at high risk in SSA [13, 19, 20] was used.

An HIV prevalence risk score was created by summing up values generated from dividing all the coefficients in the final Logistic regression model (adjusted for site) by the lowest coefficient among all the predictors (except site) in the model and rounding off to the nearest integer. Predicted HIV prevalence, area under the curve (AUC), sensitivities, specificities, positive and negative predictive values were reported at various levels of the risk score. Thereafter, we conducted 10‐fold cross validation for the risk score using the cvauroc command in Stata [21, 22] and reported the overall AUC of the 10 models developed from the internal validation, and the corresponding bootstrap bias corrected 95% CI. Similar methods were used for the model predicting HIV incidence developed from the final Poisson regression model.

### VOICE risk score validation among female participants

We then calculated female participants' modified VOICE risk scores as laboratory confirmation of STI infections required for the full risk score were unavailable [13]. We assessed the performance of the modified risk score within the PrEPVacc registration cohort by reporting one‐year HIV incidence at various levels of the risk score, calculating the AUC, Harrel's concordance index and generating Kaplan–Meier failure plots for different levels of the VOICE risk score.

## RESULTS

### HIV prevalence at screening

The Masaka site (number screened = 1359) was excluded from HIV prevalence analyses since there was pre‐screening by HIV status prior to screening for the PrEPVacc registration cohort.

A total of 2996 participants with a mean age of 26 years (SD, 5.92) were screened for participation in the registration cohort at Mbeya, Dar es Salaam, Maputo, and Durban. Of these, 2564 (86%) were female and 193 [6.4% (95% CI: 5.6–7.3)] were found to have HIV infection. Overall, most screened participants reported that they had received/given money in exchange for sex in the last month (68%), had sex after consuming alcohol in the last year (64%), had a sexual partner who was older than them by ≥10 years (63%) and that their sexual partners had other partners (82%) (Table 1).

**TABLE 1:** HIV prevalence and associated risk factors among 2996 adults screened for an HIV vaccine trial preparedness study in three African countries.

Characteristic	*N* (%)	HIV	Univariable analysis		Multivariable analysis^a^	
		Prevalence (%)	Crude OR (95% CI)*	*p* value	AdjOR (95% CI)	*p* value
	2996 (100)	193 (6.4)				
Study site, country^b^						
Dar es Salaam, Tanzania	1059 (35)	64 (6.0)	Ref		Ref	
Mbeya, Tanzania	799 (27)	46 (5.8)	0.95 (0.64–1.40)		1.42 (0.93–2.16)	
Maputo, Mozambique	552 (18)	37 (6.7)	1.12 (0.0.73–1.70)		1.49 (0.69–3.24)	
Phoenix, Durban, South Africa	235 (8)	33 (14.0)	2.54 (1.63–3.97)		3.30 (1.57–6.94)	
Verulam, Durban, South Africa	351 (12)	13 (3.7)	0.60 (0.33–1.10)	0.016	0.64 (0.29–1.41)	<0.001
Sex						
Male	432 (14)	35 (8.1)	Ref		Ref	
Female	2564 (86)	158 (6.2)	0.74 (0.51–1.09)	0.130	1.71 (0.97–3.03)	0.064
Age						
18–24 years	1474 (49)	61 (4.1)	Ref		Ref	
25–29 years	836 (28)	50 (6.0)	1.47 (1.00–2.16)		1.74 (1.16–2.60)	
30–34 years	384 (13)	41 (10.7)	2.77 (1.83–4.18)		3.36 (2.16–5.21)	
35+ years	302 (10)	41 (13.6)	3.64 (2.40–5.52)	<0.001	5.18 (3.28–8.18)	<0.001
Used recreational drugs in the last 3 months						
No	2657 (89)	160 (6.0)	Ref		Ref	
Yes	339 (11)	33 (9.7)	1.68 (1.14–2.49)	0.009	1.89 (1.22–2.94)	0.005
Unprotected anal sex with other males in the last 3 months						
No/N/A	2947 (98)	179 (6.1)	Ref		Ref	
Yes	49 (2)	14 (29.0)	6.19 (3.27–11.71)	<0.001	11.82 (4.91–28.5)	<0.001
Sex after using alcohol/recreational drugs in the last year						
No	1081 (36)	55 (5.1)	Ref		Ref	
Yes	1915 (64)	138 (7.2)	1.45 (1.05–2.00)	0.024	1.33 (0.92–1.92)	0.130
Sexual partner has other partners						
No	549 (18)	24 (4.4)	Ref		Ref	
Yes	2447 (82)	169 (6.9)	1.62 (1.05–2.51)	0.030	1.54 (0.93–2.55)	0.090
Sex partner is a long distance truck driver or miner						
No	1995 (67)	126 (6.3)	Ref		Ref	
Yes	1001 (33)	67 (6.7)	1.06 (0.78–1.45)	0.691	1.30 (0.89–1.89)	0.175
Received/gave money/goods in exchange for sex in the last month						
No	959 (32)	70 (7.3)	Ref		Ref	
Yes	2037 (68)	123 (6.0)	0.82 (0.60–1.11)	0.190	0.61 (0.33–1.14)	0.120
Diagnosed/treated for an STI in the last 3 months						
No	2778 (93)	182 (6.6)	Ref		Ref	
Yes	218 (7)	11 (5.1)	0.75 (0.41–1.42)	0.385	0.71 (0.37–1.36)	0.304
Sexual partner older by 10 years or more						
No	1094 (37)	70 (6.4)	Ref		Ref	
Yes	1902 (63)	123 (6.5)	1.01 (0.75–1.37)	0.942	0.89 (0.57–1.40)	0.619
Unprotected sex with 2 or more partners in the last 3 months						
No	828 (28)	48 (5.8)	Ref		Ref	
Yes	2168 (72)	145 (6.7)	1.16 (0.83–1.63)	0.375	1.12 (0.71–1.76)	0.619

^a^ At multivariable analysis, all predictors were adjusted for site, sex, age, recreational drug use, unprotected anal sex with other males, sex after using alcohol/drugs, sexual partner has other partners, sex partner is a long distance truck driver/miner, and receiving/giving money/goods in exchange for sex.

^b^ The Masaka, Uganda site was excluded from the prevalence analyses since there was pre‐screening prior to screening for the vaccine trial preparedness study.

### Factors associated with HIV prevalence

The following variables were significantly associated with HIV prevalence: older than age 18–24 years; 25–29 years [adjusted odds ratio (AdjOR) = 1.74, 95% CI: (1.16–2.60)]; 30–34 years [AdjOR = 3.36, 95% CI: (2.16–5.21)], ≥35 years [AdjOR = 5.18, 95% CI: (3.28–8.18)]; using recreational drugs in the last 3 months [AdjOR = 1.89, 95% CI: (1.22–2.94)], and unprotected anal sex with other males in the last 3 months [AdjOR = 11.82, 95% CI: (4.91–28.50)]. There was weak evidence that female sex [AdjOR = 1.71, 95% CI: (0.97–3.03)] and having a sexual partner who had other partners [AdjOR = 1.54, 95% CI: (0.93–2.55)] were associated with HIV prevalence. The final prevalence Logistic regression model had a Hosmer Lemeshow goodness of fit chi square (with 11 groups, df = 9) of 9.60, *p* value = 0.384, indicative of good fit.

### Creation and validation of the HIV prevalence risk score

**FIGURE 1:**
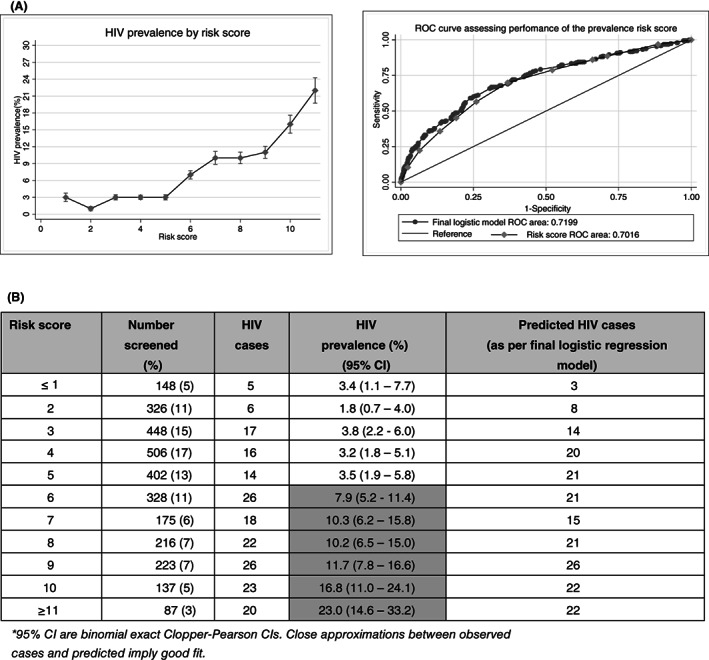
Performance of an HIV prevalence risk score. (A) AUC: Overall 0.70 (0.66–0.74); mean AUC (CI) (10‐fold cross validation) 0.70 (0.63–0.72); AUC by study site: Dar es Salaam, Tanzania 0.69 (0.63–0.75); Mbeya, Tanzania 0.67 (0.59–0.75); Maputo, Mozambique 0.77 (0.69–0.86); Durban, South Africa 0.75 (0.66–0.84) Verulam, South Africa 0.60 (0.43–0.77). *95% CI are binomial exact Clopper‐Pearson CIs. Close approximations between observed cases and predicted imply good fit. Final risk score: being female = 2 points, belonging to a particular age group (25–29 years = 2 points, 30–34 years = 5 points, 35+ years = 6 points), unprotected anal sex with other men in the last 3 months = 10 points, recreational drug use in the last 3 months = 2 points, sex after using alcohol/recreational drugs in the last 12 months = 1 point, sexual partner(s) who had other partners = 2 points, reported transactional sex = −2 points, partner who is a long distance truck driver/miner = 1 point. 97% of participants in our study had a score of <11 points. All scores ≥11 points were grouped into one category.

We then created and internally validated a risk score identifying participants more likely to have prevalent HIV infection (Figure 1; Table S2; Figure S1). The risk score had good predictive ability with an AUC of 0.70 (95% CI, 0.66–0.74). Results from the 10‐fold cross validation showed good predictive ability as well as an overall AUC and corresponding bootstrap bias corrected 95% CI of 0.70 (95% CI, 0.63–0.72).

The most suitable cut off with the highest Youden's index (Sn + SP‐1) within the prevalence risk score among the screened participants was ≥6 points. At this cut‐off, the risk score identified 70% of all positive cases within 39% of all screened [Sensitivity = 70%; Specificity = 63%] (Table S3). Additional multivariable prevalence analyses stratified by sex are provided in Table S4.

### HIV incidence

The overall HIV incidence was 2.9/100 person‐years (95% CI, 2.4–3.5) (Table 2). HIV incidence varied over time in follow‐up, with higher HIV incidence during participants' second year of follow‐up [3.6/100 person‐years (95% CI, 2.6–4.9)] than the first year of follow‐up [2.8/100 person‐years (95% CI, 2.2–3.6)]. Few participants had data beyond 2 years of follow‐up and HIV incidence seemed to fall beyond 2 years [1.4/100 person‐years (95% CI, 0.6–3.2)]. HIV incidence also varied considerably by calendar period, with the highest incidence seen in 2018 [3.3/100 person‐years (95% CI, 1.5–7.3)] and 2019 [4.1/100 person‐years (95% CI, 3.1–5.4)]. HIV incidence in the combined calendar periods of 2020 and 2021 was 2.1/100 person‐years (95% CI, 1.6–2.8). The unadjusted HIV incidence varied considerably between study sites.

**TABLE 2:** Trends in HIV incidence and risk factors for HIV acquisition among 2956 adults enrolled in a vaccine trial preparedness study in four African countries.

Characteristic		HIV incidence			Univariable analysis		Multivariable analysis^a^	
	*N* (%)	*n*	PYO	IR/100 PYO (95% CI)	Crude RR (95% CI)*	*p* value	AdjRR (95% CI)	*p* value
Overall	2956 (100)	105	3678.1	2.9 (2.4–3.5)				
Time in follow‐up								
0.00–1.00 years	N/A	62	2217.0	2.8 (2.2–3.6)	Ref		Ref	
1.01–2.00 years	N/A	37	1036.4	3.6 (2.6–4.9)	1.28 (0.85–1.92)		2.13 (1.32–3.45)	
2.01–3.20 years	N/A	6	422.2	1.4 (0.6–3.2)	0.51 (0.22–1.17)	0.065	1.04 (0.41–2.62)	0.005
Calendar period								
2018	N/A	6	184.0	3.3 (1.5–7.3)	0.80 (0.34–1.86)		0.79 (0.33–1.86)	
2019	N/A	51	1248.2	4.1 (3.1–5.4)	Ref		Ref	
2020/2021	N/A	48	2243.4	2.1 (1.6–2.8)	0.52 (0.35–0.78)	0.006	0.39 (0.24–0.62)	<0.001
Site								
Dar es Salaam, Tanzania	900 (30)	35	1551.2	2.3 (1.6–3.1)	Ref		Ref	
Masaka, Uganda	804 (27)	22	832.7	2.6 (1.7–4.0)	1.17 (0.69–2.00)		1.27 (0.63–2.56)	
Mbeya, Tanzania	567 (19)	34	706.4	4.8 (3.4–6.7)	2.13 (1.33–3.42)		1.68 (0.98–2.88)	
Maputo, Mozambique	256 (9)	5	344.1	1.5 (0.6–3.5)	0.64 (0.25–1.64)		0.92 (0.28–3.00)	
Phoenix/Verulam, Durban, South Africa	429 (15)	9	243.7	3.8 (1.9–7.1)	1.64 (0.79–3.40)	0.010	2.54 (0.75–8.61)	0.140
Gender								
Male	604 (20)	6	726.4	0.8 (0.4–1.8)	Ref		Ref	
Female	2352 (80)	99	2951.7	3.4 (2.8–4.1)	4.06 (1.78–9.26)	0.001	6.27 (2.23–17.64)	0.001
Age								
≤24	1652 (56)	63	1979.1	3.2 (2.5–4.1)	Ref		Ref	
>24	1304 (44)	42	1699.0	2.5 (1.8–3.3)	0.78 (0.53–1.15)	0.204	0.84 (0.57–1.25)	0.390
Occupation								
Other (professional, sales)	926 (31)	15	877.0	1.7 (1.0–2.8)	Ref		Ref	
Female sex worker	1612 (55)	59	2249.2	2.6 (2.0–3.4)	1.53 (0.87–2.70)		1.11 (0.39–3.18)	
Salon/lodge/bar worker	329 (11)	27	400.4	6.7 (4.6–9.8)	3.94 (2.10–7.41)		2.67 (1.02–6.97)	
Subsistence fisheries worker	89 (3)	4	151.5	2.6 (1.0–7.0)	1.54 (0.51–4.65)	<0.001	4.28 (1.10–16.61)	0.002
Education level								
≤Primary	1332 (45)	59	1952.1	3.0 (2.3–3.9)	Ref		Ref	
≥Secondary	1624 (55)	46	1726 0.0	2.7 (2.0–3.6)	0.88 (0.60–1.30)	0.522	0.82 (0.54–1.24)	0.346
Marital status								
Single	1859 (63)	73	2448.1	3.0 (2.4–3.8)	Ref		Ref	
In relationship/married/cohabiting	701 (24)	15	752.4	2.0 (1.2–3.3)	0.67 (0.38–1.17)		0.74 (0.38–1.44)	
Divorced/separated/widowed	396 (13)	17	477.6	3.6 (2.2–5.7)	1.19 (0.70–2.02)	0.237	1.07 (0.61–1.86)	0.634
Baseline behavioural risk (last 3 months)								
Used a condom at last sex								
No	2149 (73)	76	2554.4	3.0 (2.4–3.7)	Ref		Ref	
Yes	807 (27)	29	1123.7	2.6 (1.8–3.7)	0.87 (0.57–1.33)	0.515	1.00 (0.65–1.56)	0.983
Had transactional sex								
No	692 (23)	17	723.6	2.4 (1.5–3.8)	Ref		Ref	
Yes	2264 (77)	88	2954.5	3.0 (2.4–3.7)	1.27 (0.75–2.13)	0.370	1.05 (0.49–2.26)	0.892
Has anonymous/casual sex partners								
No	356 (12)	9	349.2	2.6 (1.3–5.0)	Ref		Ref	
Yes	2600 (88)	96	3328.9	2.9 (2.4–3.5)	1.12 (0.57–2.22)	0.747	1.20 (0.52–2.76)	0.670
Has partner older by 10 years								
No	986 (33)	32	1127.4	2.8 (2.0–4.0)	Ref		Ref	
Yes	1970 (67)	73	2550.7	2.9 (2.3–3.6)	1.01 (0.67–1.53)	0.969	0.76 (0.46–1.25)	0.278
Used recreational drugs								
No	2563 (87)	88	3241.4	2.7 (2.2–3.3)	Ref		Ref	
Yes	393 (13)	17	436.7	3.9 (2.4–6.3)	1.43 (0.85–2.41)	0.174	2.09 (1.20–3.65)	0.009
Had sex after consuming alcohol								
No	1008 (34)	39	1272.4	3.1 (2.2–4.2)	Ref		Ref	
Yes	1948 (66)	66	2405.7	2.7 (2.2–3.5)	0.90 (0.60–1.33)	0.583	0.75 (0.49–1.14)	0.177
Number of partners in the last 3 months at baseline								
≤5	1228 (42)	41	1360.0	3.0 (2.2–4.1)	Ref		Ref	
≥6	1728 (58)	64	2318.1	2.8 (2.2–3.5)	0.92 (0.62–1.36)	0.660	0.98 (0.57–1.69)	0.934
Diagnosed/treated for an STI in last 3 months at baseline								
No	2457 (83)	89	3178.7	2.8 (2.3–3.4)	Ref		Ref	
Yes	499 (17)	16	499.4	3.2 (2.0–5.2)	1.14 (0.67–1.95)	0.620	1.12 (0.60–2.07)	0.724
Abnormal genital discharge (last 3 months) baseline								
No	2275 (77)	85	2933.1	2.9 (2.3–3.6)	Ref		Ref	
Yes	681 (23)	20	745.0	2.7 (1.7–4.2)	0.93 (0.57–1.51)	0.758	0.73 (0.41–1.29)	0.280
Genital ulcer (last 3 months) at baseline								
No	2652 (90)	95	3326.8	2.9 (2.3–3.5)	Ref		Ref	
Yes	304 (10)	10	351.3	2.8 (1.5–5.3)	1.00 (0.52–1.91)	0.992	1.01 (0.50–2.03)	0.983
Initiated PrEP during follow up								
No	2707 (92)	97	3244.1	3.0 (2.5–3.7)	Ref		Ref	
Yes	249 (8)	8	434.0	1.8 (0.9–3.7)	0.62 (0.30–1.27)	0.188	0.64 (0.57–1.26)	0.412

^a^ At multivariable analysis, all predictors were adjusted for site, gender, age, occupation, using recreational drugs in the last 3 months and sex after consuming alcohol. Time related predictors (time in follow up and calendar period) were in addition adjusted for one another.

HIV incidence did not vary with age (*p* value = 0.390) but was higher among female than male participants (AdjRR = 6.27, 95% CI, 2.23–17.64) and associated with participant occupation [Salon/lodge/bar worker (85% female) (AdjRR = 2.67, 95% CI, 1.02–6.97); fisher folk (AdjRR = 4.28, 95% CI, 1.10–16.61)] and recreational drug use within the last 3 months (AdjRR = 2.09, 95% CI, 1.20–3.65).

About 8% of all participants reported taking PrEP at least at one 6‐monthly assessment during follow up. PrEP uptake varied widely across sites ranging from 0% (Durban, Maputo) to 1% (Mbeya), 8% (Dar es Salaam) and 21% (Masaka). There was limited evidence of association between PrEP uptake and HIV incidence at multivariable analysis (AdjRR = 0.64, 95% CI, 0.57–1.26).

Of the 2998 participants included in this analysis, 825 (28%) were terminated from the cohort for reasons which can be considered loss to follow up: uncontactable/untraceable (268), moved from area (252), declined further follow up (299), death (6).

### HIV incidence risk score

There was limited evidence of associations between self‐reported HIV risk indicators and HIV incidence making it unfeasible to create an HIV incidence risk score based on such variables.

### VOICE risk score performance

Overall, 92% of female participants had a modified VOICE risk score ≥ 5 (expected in the original study to predict an HIV incidence of ≥5/100 person‐years) [13]. The overall HIV incidence among female participants over the first year of follow‐up was 3.6/100 person‐years (Table 3). Despite indications of a linear trend with HIV incidence, the VOICE risk score did not clearly distinguish between high and low risk participants (Figure S2) and performed poorly within our cohort (AUC = 0.58, 95% CI, 0.51–0.64; Harrell's concordance index = 0.59). We also calculated sensitivities and specificities of the VOICE risk score (Tables S5 and S6).

**TABLE 3:** Performance of the VOICE risk score within the PrEPVacc registration cohort.

Modified VOICE risk score	Female participants with follow‐up (%)	Person years of follow‐up	HIV cases	HIV incidence (per 100Pyrs) (95% CI)
Overall	2352 (100)	1858.7	66	3.6 (2.8–4.5)
≤3	74 (3)	58.0	1	1.7 (2.4–12.2)
4	106 (5)	82.3	2	2.4 (0.6–9.7)
5	284 (12)	223.3	5	2.2 (0.9–5.4)
6	777 (33)	634.1	21	3.3 (2.2–5.1)
7	361 (15)	265.3	14	5.2 (3.1–8.9)
8	750 (32)	595.8	23	3.9 (2.6–5.8)

*Note*: Modified VOICE risk score: age < 25 years = 2 points; unmarried/not living with partner = 2 points; partner does not provide financial/material support = 1 point; primary partner has other partners (yes or do not know) = 2 points; alcohol use in the last 3 months = 1 point. AUC (95% CI): 0.58 (0.51–0.64). Harrel's concordance index: 0.59.

## DISCUSSION

### HIV prevalence

The overall prevalence of HIV infection among individuals screened for the PrEPVacc registration cohort was 6.4% and varied by study site, demographic and behavioural characteristics.

HIV prevalence was higher among participants in the older age groups compared to those in the youngest age group (18–24) emphasising the need for intensified screening and linkage to care in older individuals and intensified HIV prevention interventions in younger people among populations considered to be at high risk.

Women were more likely to have prevalent HIV infection than men. This has been widely acknowledged in literature [23, 24, 25, 26, 27, 28], with some attributing the higher prevalence in women to higher biological susceptibility [29], having older partners [30, 31, 32] (in our study 73% of women reported having older partners vs. 17% of men), limited access to financial resources compared to men, and higher prevalence of other sexually transmitted infections (STIs) such as herpes simplex virus 2 (HSV‐2) [24, 25, 33].

Male participants who reported having unprotected anal sex with other males in the last 3 months were more likely to have prevalent HIV infection than heterosexual men and women, with an overall prevalence of HIV of 29% (95% CI, 16%–41%). Studies in SSA have reported HIV prevalence ranging from 12% to 45% [34, 35, 36] among MSM. Most of the men who reported unprotected anal sex in this study were from the Maputo site, Mozambique. The lack of information and HIV prevention interventions in MSM populations in African settings where same sex sexual activity is criminalised may compromise efforts to bring the HIV epidemic under effective control despite the progress in reducing HIV incidence in other populations globally [34, 35, 36].

We found a strong association between use of recreational drugs and HIV prevalence, similar to other studies in Africa [37, 38]. Over the last few years, use of recreational drugs has steadily increased in African settings [39]. HIV prevention interventions should be encouraged among individuals who use recreational drugs and report having sex while under the influence of drugs or alcohol. Individuals who reported that they had sexual partners who had other partners were also at elevated risk of HIV as reported elsewhere [26, 40].

In our study, reported transactional sex had a negative association with HIV prevalence. It is possible that individuals who report transactional sex are more aware of their risk and if HIV positive were already aware of it and thus did not present for screening.

### HIV prevalence risk score

Our HIV prevalence risk score performed well across sites with an overall AUC of 0.70 and identified 70% of all HIV prevalent cases within 39% of all participants screened when the cut‐off of ≥6 was used to identify those at higher risk. This risk score estimates an individual's relative risk of prevalent HIV infection in comparison to other individuals in their community. It does not estimate an individual's absolute risk of prevalent HIV infection, which is dependent on the community HIV prevalence in their setting. To approximate an individual's absolute risk of HIV, a function of the community prevalence and their prevalence risk score would be needed.

The prevalence risk score we developed could potentially be used to encourage HIV testing in parts of SSA, alongside routine promotion of testing for all and other HIV information dissemination efforts. Modification of the risk score to incorporate additional risk factors relevant to local contexts could further improve its performance. Such a risk score could be developed into a phone application (with inbuilt setting‐specific HIV prevalence estimates), circulated on social media or printed in local newspapers encouraging individuals with a certain risk score to get tested. In SSA, there is a notable lack of information on the utilisation of HIV prevalence data and associated factors to create risk scores that identify individuals who could have prevalent HIV infection. Our prevalence risk score was inspired by the Denver HIV risk score that is widely used for targeted HIV testing in the United States [41].

We anticipate that our risk score should be generalisable to settings and populations similar to those included in our study. Limited generalisability is expected in populations that differ considerably from ours, demographically, by HIV prevalence and by HIV epidemiology, considering that the risk factors associated with HIV infection may vary across contexts and change over time. Additionally, this analysis was carried out in populations expected to be at high risk of HIV, hence generalisability to populations at much lower risk may be limited. Future studies assessing generalisability to other settings are needed for more insight on this.

A mock HIV prevalence prediction tool has been provided in Figure S1. Modifications may be needed to ensure the negative association between HIV prevalence and reported transactional sex is not misunderstood by end users.

### HIV incidence

The observed overall HIV incidence was 2.9/100 person‐years but differed by study site. HIV incidence at univariable analysis seemed to drop in the third year of follow‐up; however, our study was partly conducted during a period of COVID‐19 related restrictions across the different settings [42] likely affecting HIV incidence in the latter period due to limited social interaction.

As observed in the prevalence data, women had significantly higher HIV incidence than their male counterparts. Similar to our findings, women working in salons, lodges or bars have previously been identified as being at high risk of HIV [28, 43]. In our study, 84% of all female participants reported sex in exchange for money or goods yet only 68% identified as sex workers. Self‐identified sex workers may be different from those that do not self‐identify in ways that could modify risk e.g., they tend to be professionals who work from designated locations while non‐professional/indirect sex workers tend to be mobile street‐side vendors, bar waitresses, students, and bar workers [44]. Self‐identified sex workers have been reported to have higher uptake of HIV prevention interventions such as condoms and PrEP than other women at high risk of HIV [18]. Recreational drug use was also strongly associated with higher HIV incidence.

### HIV incidence risk score

We were unable to create an HIV incidence prediction tool. This is partly because our cohort largely comprised individuals that were considered to be at high risk of HIV in their settings. Additionally, the differences between participants at different study sites, site‐specific norms, and the few observed infections at some sites may have added complexity.

### VOICE risk score performance

Overall, the modified VOICE risk score performed poorly among women in our study. This was likely a result of other factors not included in the modified VOICE risk score such as site, calendar period and occupation, being strongly associated with HIV incidence in our cohort and determining the distribution of the HIV sero‐conversions. The poor performance could also partially be a result of our cohort being largely comprised of individuals at high risk, in whom self‐reported risk data may no longer be strongly predictive of HIV incidence. STI and HSV2 data were generally not collected for our study so we could not validate the full VOICE risk score. Including such data may have increased the predictive ability of the score in our cohort.

### Study strengths and limitations

STI testing was only conducted at the Dar es Salaam site. STI status data across sites could have significantly improved the prevalence and incidence prediction tools had it been available. In addition, the discrepancies between the populations screened for enrolment into the cohort at the different sites may have added complexity to the development of the prevalence risk score. For some sites, there was more pre‐screening (by risk indicators and HIV status) than other sites. This could have introduced selection bias. Most affected was the Masaka site where only 7 cases were identified in 1359 participants, which we excluded from the prevalence analyses. We anticipate that the discrepancies between sites at screening affected the reported prevalence estimates though possibly having less of an effect on the associations with risk indicators.

External validation of our prevalence risk score is needed to assess its generalisability to populations outside our study setting. Studies reporting AUC, sensitivity, specificity and that assess the real‐world applicability of the risk score in other SSA settings would be valuable.

## CONCLUSION

Overall, we identified several factors that were highly associated with HIV prevalence. This enabled the creation of a tool that can be used to identify individuals at high risk of having prevalent HIV infection, encourage HIV testing and timely linkage to ART for those found to have HIV infection.

HIV incidence was associated with calendar period, study site, gender, occupation and recreational drug use. There was limited evidence of association with other HIV risk indicators and it was not possible to create an HIV incidence risk score. This is probably because all the individuals enrolled into the vaccine trial preparedness study were at high risk of acquiring HIV based on their self‐reported behavioural risk indicators. For better prediction of HIV incidence in similar populations, more objective measures such as STI status may be needed.

## FUNDING INFORMATION

The PrEPVacc registration cohort study is funded by The Second European & Developing Countries Clinical Trials Partnership (EDCTP2) (Grant number: RIA‐2016V‐1644). RH receives funding from the UK Medical Research Council (MRC) and the UK Foreign, Commonwealth and Development Office (FCDO) under the MRC/FCDO Concordat agreement and is also part of the EDCTP2 programme supported by the European Union (Grant Ref: MR/R010161/1).

## ETHICS STATEMENT

Ethical approval was obtained from the London School of Hygiene & Tropical Medicine ethics committee, Imperial College Research Ethics Committee, and the research ethical committees for all the different study sites.

## INFORMED CONSENT

Informed consent to participate and for publication, was obtained from all the study participants.

## Supporting information


**Figure S1.** A mock template of the HIV prevalence risk score.
**Figure S2.** Performance of the VOICE risk score within the PrEPVacc registration cohort.
**Table S1.** Study variables included at analysis.
**Table S2.** Risk score as per the prevalence analysis's final model.
**Table S3.** Sensitivities, specificities, PPV and NPV of the prevalence risk score.
**Table S4.** Multivariable HIV prevalence analysis and risk scores by sex.
**Table S5.** VOICE risk score.
**Table S6.** Performance of the VOICE risk score.

## Data Availability

The datasets generated and/or analysed during the current study have been made available on the LSHTM data compass repository (https://doi.org/10.17037/DATA.00003540).
