# Supplementary material for: Developing HIV risk prediction tools in four African settings
Source: Trop Med Int Health. 2023 Jul 27;28(9):720–30. doi: 10.1111/tmi.13916 (PMC10947046; doi:10.1111/tmi.13916)
Supplement: Supplementary file 1 — Figure S1. A mock template of the HIV prevalence risk score. Figure S2. Performance of the VOICE risk score within the PrEPVacc registration cohort. Table S1. Study variables included at analysis. Table S2. Risk score as per the prevalence analysis's final model. Table S3. Sensitivities, specificities, PPV and NPV of the prevalence risk score. Table S4. Multivariable HIV prevalence analysis and risk scores by sex. Table S5. VOICE risk score. Table S6. Performance of the VOICE risk score. [file TMI-28-720-s001.docx]

**Supplementary table 1: Study variables included at analysis.**

| ***Key Study variables (collected at baseline, available for all screened participants)*** |
| --- |
| **Main outcome:** HIV status at screening  **Predictors:**  Study site, country (Dar es Salaam, Tanzania, Masaka, Uganda, Mbeya, Tanzania, Maputo, Mozambique, Durban/ Verulam, South Africa)  Sex (M/F)  Age (18 – 24, 25 – 29, 30 – 34, 35 + years)  Sex after consuming alcohol or using recreational drugs in the last year (N/Y)  Unprotected anal sex with other males in the last 3 months (No/NA/ Yes)  Used recreational drugs (including via injection) in the last 3 months (No/ Yes)  Sexual partner older by 10 years or more (N/ Y)  Sexual partner has other partners (N/Y)  Sex without a condom with 2 or more partners in the last 3 months? (N/Y)  Received or giving money/ goods in exchange for sex in the last month (N/Y)  Diagnosed/ treated for an STI in the last 3 months (N/Y)  Current abnormal genital discharge or ulcer or other genital symptoms? (N/Y)  Abnormal genital discharge or genital ulcers or other genital symptoms in the last 3 months? (N/Y)  Sex without a condom with a partner who is a long-distance truck driver or miner in the last 6 months? (N/Y) |

*Once enrolled into the cohort, additional demographic and behavioural risk data was collected from study participants.

| ***Key study variables (available for all participants in the incidence analysis)*** |
| --- |
| **Main outcome:** HIV acquisition within a maximum follow-up period of 3 years  **Predictors:**  Time in follow-up (0 - 1 years/ 1.01- 2.00/ 2.01-3.20 years)  Calendar period (2018/2019 /2020-2021) *  Site (Dar es Salaam, Tanzania/ Masaka, Uganda/ Mbeya, Tanzania/ Maputo, Mozambique/ Durban/Verulam, South Africa)  Sex (M/F)  Age (≤24, >24)  Occupation (Other/ Sex worker/ Salon/Lodge/Bar worker/ Subsistence fisheries worker)  Education level (≤ Primary, ≥Secondary)  Marital status (Single or (divorced/widowed/separated) or (in a relationship/ married/cohabiting))  **(Baseline Behavioral risk (last 3 months))**  Used a condom at last sex (Y/N)  Had transactional sex in the last 3months (Y/N)  Has anonymous/ casual sex partners (Y/N)  Has partner older by 10 years (Y/N)  Used recreational drugs in the last 3 months (Y/N)  Had Sex after consuming alcohol (Y/N)  Number of partners last 3months (≤5, ≥6)  Diagnosed/ treated for an STI in last 3 months (Y/N)  Abnormal genital discharge (last 3 months) (Y/N)  Genital ulcer (last 3 months) (Y/N)  Initiated PrEP during follow up (Y/N) |

**2020 and 2021 were combined into one calendar period at analysis due to limited follow up time for 2021.*

**Supplementary table 2: Risk score as per the prevalence analysis’s final model**

| **Variable** |  | **Coefficient (β)** | **Risk score** | |
| --- | --- | --- | --- | --- |
| Sex of participant | Female | .538157 | 2 |  |
| Age of participant | 18 – 24 years | Ref |  |  |
|  | 25 – 29 years | .5527923 | 2 |  |
|  | 30 - 34 years | 1.211378 | 5 |  |
|  | 35-45 years | 1.644799 | 6 |  |
| If male, unprotected anal sex with other men in the last 3 months | Yes | 2.469541 | 10 |  |
| Used recreational drugs (injectable or non-injectable) in the last 3 months | Yes | .6372189 | 2 |  |
| Sex after consuming alcohol or using recreational drugs in the last 12 months | Yes | .2845902 | 1 |  |
| Partner has other partners | Yes | .4339203 | 2 |  |
| Participant reported having transactional sex in last 3 months | Yes | -.4966063 | -2 |  |
| Partner is a long distance truck driver or miner | Yes | .2596882 | 1 |  |

*Smallest predictor: .2596882

**Supplementary figure 1: A mock template of the HIV prevalence risk score.**

**
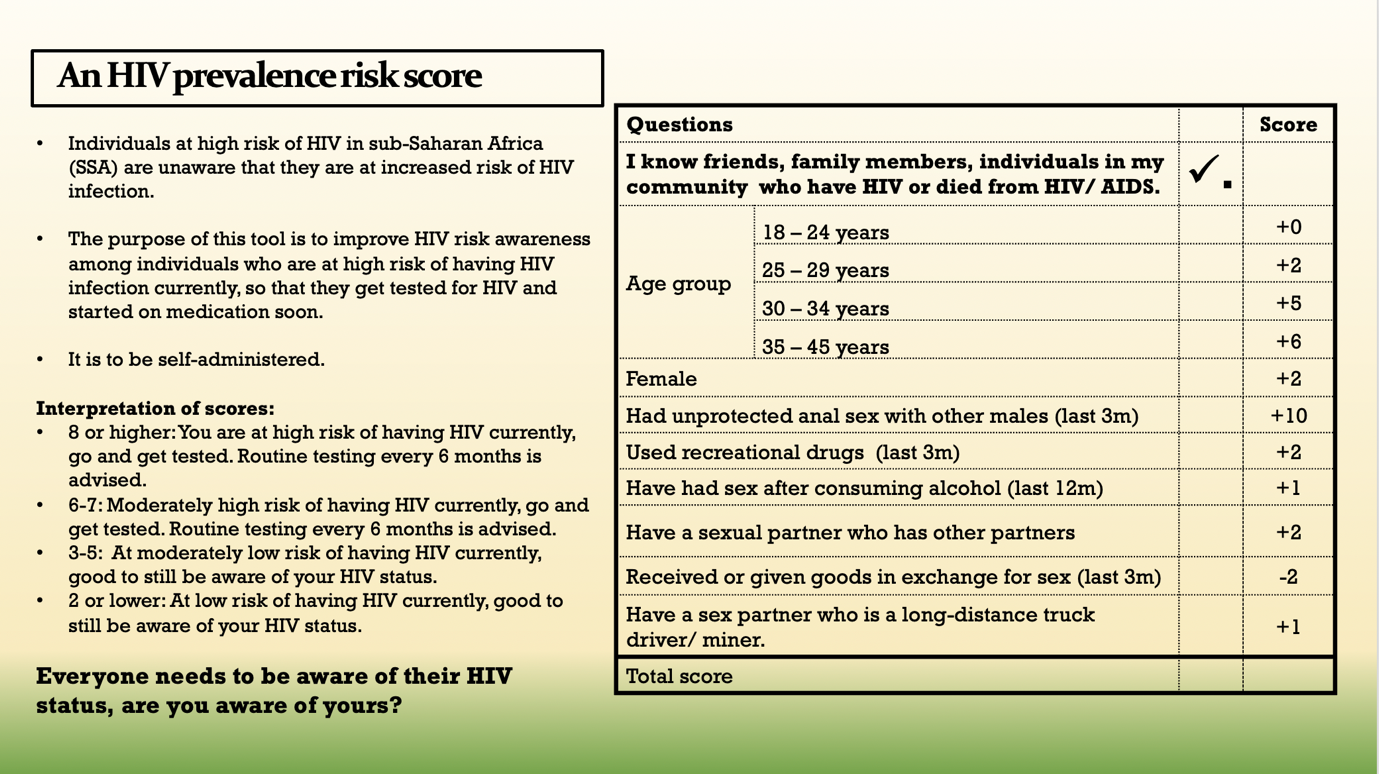
**

We identified factors predictive of prevalent HIV infection among individuals considered to be at high risk of HIV in SSA, and combined them into a risk score. This tool is aimed at increasing efficiency in early diagnosis of HIV infection, such that individuals who already have HIV can get tested, and get started on Anti-retroviral therapy. This tool estimates an individual’s relative risk of prevalent HIV infection in comparison to other individuals in their community. It does not estimate an individual’s absolute risk of prevalent HIV infection, which is dependent on the community HIV prevalence in their setting. It also does not predict likelihood of getting HIV in the future.

[Modifications may be needed to ensure the negative association between HIV prevalence and reporting transactional sex is not misunderstood by end users].

**Supplementary table 3: Sensitivities, Specificities, PPV and NPV of the prevalence risk score**

| **Risk score cut offs** | **Participants in the higher risk group (%) as per cutoff** | **Sensitivity (%)** | **Specificity (%)** | **PPV**  **/ HIV prevalence in higher risk group (%)** | **NPV (%)** | **Youden’s index (%)** |
| --- | --- | --- | --- | --- | --- | --- |
| ≥ 1 | 2996 (100) | 100 | 0 | 6.4 | N/a |  |
| ≥ 2 | 2848 (95) | 97 | 5 | 6.6 | 97.6 | 2 |
| ≥ 3 | 2522 (84) | 94 | 17 | 7.2 | 97.7 | 11 |
| ≥ 4 | 2074 (69) | 85 | 32 | 8.0 | 97.0 | 17 |
| ≥ 5 | 1568 (52) | 77 | 49 | 9.5 | 96.9 | 26 |
| ≥ 6 | 1166 (39) | 70 | 63 | 11.6 | 96.8 | 33 |
| ≥ 7 | 838 (28) | 56 | 74 | 13.0 | 96.1 | 30 |
| ≥ 8 | 663 (22) | 47 | 80 | 13.7 | 95.6 | 27 |
| ≥ 9 | 447 (15) | 36 | 87 | 15.4 | 95.1 | 23 |
| ≥ 10 | 224 (7) | 22 | 94 | 19.2 | 94.6 | 16 |
| ≥ 11 | 87 (3) | 10 | 98 | 23.0 | 94.0 | 8 |

*PPV: Positive predictive value; NPV: Negative predictive value.*

*The most suitable cut off within the participants screened was ≥6. It identified 70% of all positive cases within 39% of all screened.*

**Supplementary table 4: Multivariable HIV prevalence analysis and risk scores by sex**

**Males only (multivariable analysis and prevalence risk score)**

|  |  | **HIV** | **Multi-variable analysis¥&** | | |
| --- | --- | --- | --- | --- | --- |
| **Characteristic** | **N (%)** | **Prevalence (%)** | **AdjOR (95% CI)** | **P-value** | **Risk score** |
|  | **432 (100)** | **35 (8.1)** |  |  |  |
| Study site, country |  |  |  |  |  |
| Durban, South Africa | 103 (24) | 12 (11.7) | Ref |  |  |
| Maputo, Mozambique | 250 (58) | 22 (8.8) | 0.49 (0.20 – 1.23) |  |  |
| Verulam, South Africa | 79 (18) | 1 (1.3) | 0.10 (0.01 – 0.82) | 0.054 |  |
| Age  18 – 24 years  25 – 29 years  30 – 34 years  35+ years |  |  |  |  |  |
|  | 276 (64) | 14 (5.1) | Ref |  |  |
|  | 94 (22) | 8 (8.5) | 3.07 (1.10 – 8.58) |  | 1 |
|  | 44 (10)  18 (4) | 8 (18.2)  5 (27.8) | 7.77 (2.56 – 23.62)  13.88 (3.65 – 52.83) | <0.001 | 2  2 |
| Unprotected anal sex with other males in the last 3 months |  |  |  |  |  |
| No | 383 (89) | 21 (5.5) | Ref |  |  |
| Yes | 49 (11) | 14 (28.6) | 13.37 (4.93 – 36.28) | <0.001 | 2 |

***** *At multivariable analysis, forward Stepwise logistic regression was used. Predictors were added to the model if they had a P-value<0.2.* ***AUC = 0.81 (0.74 – 0.89) Risk score AUC = 0.78 (0.69 – 0.86)**

**Females only (Multivariable HIV prevalence analysis and risk score)**

|  |  | **HIV** | **Multi-variable analysis¥** | | |
| --- | --- | --- | --- | --- | --- |
| **Characteristic** | **N (%)** | **Prevalence (%)** | **AdjOR (95% CI)** | **P-value** | **Risk score** |
|  | **2564 (100)** | **158 (6.2)** |  |  |  |
| Study site, country |  |  |  |  |  |
| Dar es Salaam, Tanzania | 1059 (41) | 64 (6.0) | Ref |  |  |
| Mbeya, Tanzania | 799 (31) | 46 (5.8) | 1.41 (0.92 – 2.16) |  |  |
| Maputo, Mozambique | 302 (12) | 15 (5.0) | 1.21 (0.47 – 3.09) |  |  |
| Durban, South Africa | 132 (5) | 21 (15.9) | 3.28 (1.32 – 8.12) |  |  |
| Verulam, South Africa | 272 (11) | 12 (4.4) | 0.64 (0.26 – 1.56) | <0.001 |  |
| Age  18 – 24 years  25 – 29 years  30 – 34 years  35+ years |  |  |  |  |  |
|  | 1198 (47) | 47 (3.9) | Ref |  |  |
|  | 742 (29) | 42 (5.7) | 1.57 (1.02 – 2.43) |  | 2 |
|  | 340 (13)  284 (11) | 33 (9.7)  36 (12.7) | 3.04 (1.88 – 4.92)  4.61 (2.82 – 7.53) | <0.001 | 4  6 |
| Used recreational drugs in the last 3 months |  |  |  |  |  |
| No | 2328 (91) | 131 (5.6) | Ref |  |  |
| Yes | 236 (9) | 27 (11.4) | 2.35 (1.46 – 3.78) | <0.001 | 3 |
| Sexual partner has other partners |  |  |  |  |  |
| No | 350 (14) | 12 (3.4) | Ref |  |  |
| Yes | 2214 (86) | 146 (6.6) | 2.19 (1.14 – 4.23) | 0.019 | 3 |
| Sex partner is a long distance truck driver or miner |  |  |  |  |  |
| No | 1572 (61) | 91 (5.8) | Ref |  |  |
| Yes | 992 (39) | 67 (6.8) | 1.38 (0.94 – 2.01) | 0.101 | 1 |
| Received/gave money/goods in exchange for sex in the last month |  |  |  |  |  |
| No | 585 (23) | 42 (7.2) | Ref |  |  |
| Yes | 1979 (77) | 116 (5.9) | 0.51 (0.23 – 1.16) | 0.107 | -2 |
| Sex after using alcohol/recreational drugs in the last year |  |  |  |  |  |
| No | 890 (35) | 42 (4.7) | Ref |  |  |
| Yes | 1674 (65) | 116 (6.9) | 1.31 (0.87 – 2.0) | 0.195 | 1 |

***** *Final model was adjusted for site, age, recreational drug use, sexual partner has other partners, Sex partner is a long distance truck driver or miner, Received/gave money/goods in exchange for sex in the last month, and sex after using alcohol/recreational drugs in the last year* ***AUC = 0.70 (0.66-0.75) Risk score AUC = 0.68 (0.64 – 0.72)**

| **Modified VOICE risk score** | **Risk score** |
| --- | --- |
| Age (< 25) | 2 |
| unmarried or not living with partner | 2 |
| Partner does not provide financial or material support | 1 |
| Primary partner has other partners (yes or don’t know) | 2 |
| Alcohol use in the last 3 months | 1 |
| Maximum score | 8 |

**Supplementary table 5: VOICE risk score**

**Supplementary table 6: Performance of the VOICE risk score.**

| **VOICE risk score cut off** | **Number of participants in higher group (%)** | **Sensitivity (%)** | **Specificity (%)** | **PPV**  **/ new sero conversions in higher risk group over 1 year** | **NPV** |
| --- | --- | --- | --- | --- | --- |
| ≥ 5 | **2172 (92)** | **95** | **8** | **2.9** | **98.3** |
| ≥ 6 | 1888 (80) | 88 | 20 | 3.1 | 98.3 |
| ≥ 7 | 1111(47) | 56 | 53 | 3.3 | 97.7 |
| ≥ 8 | 750 (32) | 35 | 68 | 3.1 | 97.3 |

*PPV: Positive predictive value; NPV: Negative predictive value. Overall sero-conversion: 66/2352 (2.8%)*

**Supplementary figure 2: Performance of the VOICE risk score within the PrEPVacc registration cohort**

**The category of participants who had a VOICE risk score of 7, were mostly single women, aged less than 25years, evenly distributed across study sites, with a higher proportion from the Mbeya site (38%).*
